# Can EAT be an INOCA goalkeeper

**DOI:** 10.3389/fendo.2022.1028429

**Published:** 2023-01-20

**Authors:** Tong Shan, Zheng Shuwen, Wu Hengbin, Zeng Min

**Affiliations:** ^1^ Center of Geriatrics, Hainan General Hospital (Hainan Affiliated Hospital of Hainan Medical University), Haikou, China; ^2^ Clinical College, Hainan Medical University, Haikou, China

**Keywords:** epicardial adipose tissue, coronary microvascular dysfunction, ischemic non-obstructive coronary artery disease, adipocytokine, inflammatory mediators

## Abstract

Ischemia with non-obstructive coronary artery (INOCA) is a blind spot of coronary artery disease (CAD). Such patients are often reassured but offered no specific care, that lead to a heightened risk of adverse cerebrovascular disease (CVD) outcomes. Epicardial adipose tissue (EAT) is proven to correlate independently with CAD and its severity, but it is unknown whether EAT is a specific and sensitive indicator of INOCA. This review focuses on the INOCA epidemiology and related factors, as well as the association between EAT.

## 1 Background

Ischemia with non-obstructive coronary artery (INOCA) has become the focus of global attention. In tradition myocardial ischemia is caused by coronary atherosclerosis-induced obstructive lesions. However, many patients exhibit chest pain and ischemia without significant vascular disease (≥50%) (1). Such patients are often neglected, resulting in frequent hospitalization and adverse clinical outcome (2–6). The mechanisms of INOCA are multifactorial, including coronary microvascular dysfunction (CMD), coronary vascular spasm, endothelial dysfunction, myocardial bridge, both alone or co-exist (7). CMD may be the main reason, but it is difficult to detect sensitivity (3–6). Epicardial adipose tissue (EAT) occupies a special place within the heart. During the progression of atherosclerosis (AS), the paracrine function of EAT changes from a nutritional source to lesions with dysfunction, inflammation, and fibrosis (8). We suspect that the thickness or density of EAT could become a new diagnostic criterion for INOCA. Extensive studies have confirmed that EAT is not only independently associated with the severity of CAD but also with the plaque calcification (9). However, others have some inconsistent and women with CAD are susceptible (5, 10, 11). This illustrates whether these differences are early features of CAD, while EAT is a sensitive predictor for INOCA. This review focuses on INOCA epidemiology and related factors, as well as its association between EAT.

## 2 Ischemia with Non-obstructive Coronary Artery (INOCA)

INOCA is characterized by clinical evidence of myocardial ischemia with either normal or less than 50% stenotic coronary arteries on angiography. Causes of INOCA include CMD, coronary vascular spasm, endothelial dysfunction, where CMD is the main (50%). Traditionally invasive and noninvasive CMD detection have corresponding blind spots: blockage under invasive coronary angiography may have no dysfunction, and FFR <0.8 may be overrated.

### 2.1 INOCA and CMD

Balaji T. found that approximately half of the women with INOCA had CMD (age 57 ± 11; MPRI <1.84) (2). CMD is a disease with injured epicardium endothelium, limiting perfusion of the myocardium. It is characterized by impaired coronary flow reserve (CFR), which may be due to obstructive or nonobstructive CAD and myocardial infarction. Usually, CMD has a relatively long asymptomatic period, but 30-60% can both incite angina (6). As reflected in CFR, there is little correlation between anatomical severity and functional impairment. Thus, CMD should be recognized as the main cause of INOCA.

Among the sites controlling blood flow resistance and perfusion is the coronary artery microcirculation (diameter<300 mm), including arterioles and small veins. A major part of circulation is the delivery of oxygen and nutrients to tissues, the removal of waste and the regulation of inflammation and repair. Because of the adaptation of their morphology and function, R Pries proposed the model of “vascular adaptation” to describe microvascular network adaptation in meeting tissue requirements in 1998. The adaptation of the vascular cavity to the circulatory system ensures that the vascular cavity and all tissues are equally perfused and thus ensures adequate perfusion reserves and good perfusion at rest. Nevertheless, microcirculation is poorly adapted, resulting in insufficient blood flow distribution and degradation of microcirculation.

**Figure 1:**
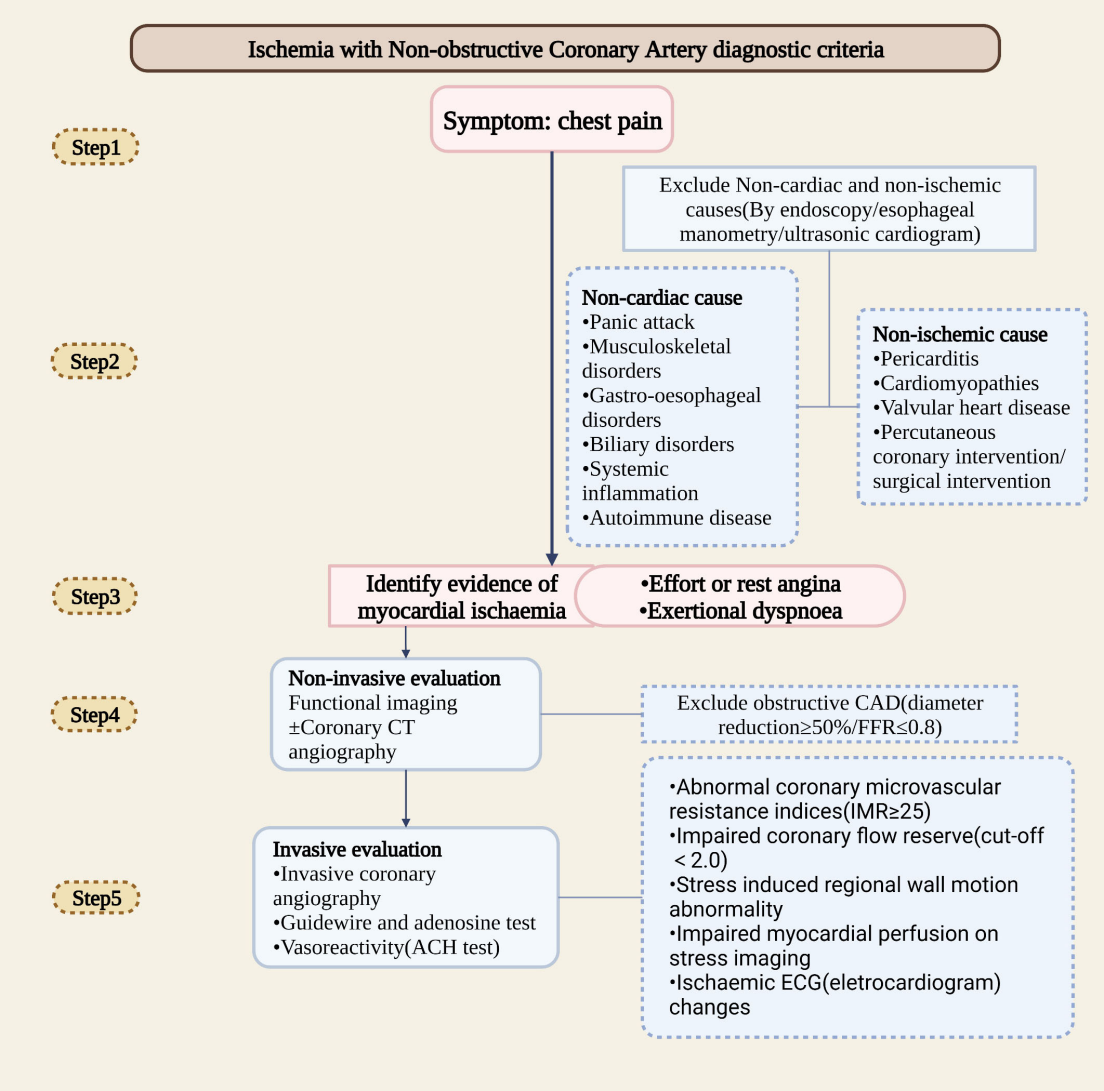
The clinical diagnostic assessment of INOCA. (Created with BioRender.com).

In clinical, myocardial ischemia mainly caused by damage of the epicardial coronary artery and downstream microvascular network (4). Total perfusion in the larger myocardial area is controlled by the epicardial artery. Due to the flow limitation, there will be a mismatch between myocardial supply and demand, thereby increasing the arteriovenous oxygen difference (AVD-O_2_). Within the terminal vascular network, microcirculation controls blood flow distribution. Microvascular dysfunction is usually associated with a functional shunt, hypoxia, and an average AVD-O_2_ reduction (12). Additionally, diffuse nonobstructive coronary AS is often underestimated since uneven distribution of microvessel results in less sensitivity of clinical imaging methods to detect heterogeneity (4). According to studies, patients with limited nonobstructive diseases have a worse prognosis than those with normal coronary arteries but better prognosis than those with extensive nonobstructive diseases. In patients with extensive nonobstructive disease, risk equals those with single-vessel obstructive CAD (13). The breakthrough for detecting INOCA early may be the sensitive detection of diffuse nonobstructive coronary AS. The clinical diagnostic assessment of INOCA is as followed (**Figure1**).

### 2.2 Diagnostic assessment of INOCA

Electrocardiograms can be used to detect myocardial ischemia in the diagnosis of INOCA. Cardiac magnetic resonance (CMR) and pressure echocardiography (left anterior descending arterial flow imaging) detected abnormal wall movement. Additionally, myocardial contrast ultrasound, single-photon emission CT (SPECT), and positron emission tomography (PET) can detect changes in perfusion (**Table 1**). Ischemia is usually caused by exercise or drugs that increase myocardial oxygen demand or by imbalanced perfusion of the heart due to vasodilation. In clinical, adenosine injections were used to measure the ratio between distal coronary artery stenosis and aortic root pressure. A flow reserve fraction (FFR) less than 0.8 indicates myocardial ischemia. In INOCA patients, customized treatment guided by the results of intracoronary trials [CFR, index of microcirculatory resistance (IMR), and acetylcholine trials] (IMR ≥ 25 units or CFR<2.32 suggests microcirculation abnormalities, is the best predictor) can significantly reduce angina symptoms (5). With the use of vasodilators such as adenosine, CFR and IMR are commonly measured. For testing the arterioles, however, acetylcholine must be injected into the coronary arteries to ascertain endothelial function. During reactive hyperemia, systemic endothelial dysfunction can be observed in INOCA patients (1).

**Table 1:** Comparison of methods for detecting INOCA.

The author	Year	Sample future and size	Method	Sensitivity	Specificity
(14) Joanne D. Schuijf, PhD	2020	381, Mean age 62 (56–68)	CT angiography /CT perfusion	Stenosis ≥50% (70%), Stenosis ≤50% (25%)	
			ICA/SPECT	Stenosis ≥50% (57%), Stenosis ≤50% (17%)	
(15) Juhani Knuuti a meta-analysis	2018	META analysis A total of 28,664 patients from 132 studies that used ICA as reference and 4,131 from 23 studies using FFR	Stress ECG Stress echo CCTA SPECT PET Stress CMR	58% 85% 97% 87% 90% 90%	62% 82% 78% 70% 85% 80%
(16) Carl J. Pepine, MD	2010	189 women, Mean age (55±10)	adenosine based CFR measurements, CFR < 2.32	62%	65%
(17) Amir Ahmadi, MD	2018	254 (64% male), Mean age (64±10)	CTA-based high-risk plaques and FFR	(non-HRPs) 96%-100%	
(18) Haseeb Rahman	2021	75, Mean age (57±10)	CMR (3-T), stress MBF, transmural MPR: MBF_HYPEREMIA_/ MBF_REST_, subendocardial MPR	41%	58%
(19) Louise E J Thomson	2015	118 women, Mean age (53.9±11.4)	cardiac magnetic resonance imaging (CMRI), myocardial perfusion reserve index (MPRI) threshold of 1.84	73%	74%
(20) Thomas J Ford	2018	151, Mean age 61.0 (53-68)	Invasive coronary artery function testing	88.7%	64%-77%

CCTA, coronary computed tomography angiography; SPECT, single-photon emission computed tomography; PET, positron emission tomography;

CFR, Coronary flow reserve; CMR, cardiac magnetic resonance; MBF, myocardial blood flow; MPR, myocardial perfusion reserve.

An intravenous injection of contrast agent was used to evaluate the coronary artery lumen wall by coronary artery CTA. Since both tests are anatomically based, it provides a high degree of accuracy for detecting coronary artery stenosis. A stenosis of 50-90% is not necessarily diagnostic, as it does not always cause myocardial ischemia (14, 21). Coronary computed tomography angiography (CCTA) is recommended as a primary or secondary diagnosis for patients at moderate risk of CAD, which due to its sensitivity and specificity (15). CCTA can be used as a pre-diagnostic tool to detect early stages of AS in healthy or mildly symptomatic patients. The 2021 ESC Guide noted that CCTA is used to exclude CAD. For clinically ST-segment elevation angina, invasive coronary angiography (ICA) will continue to be the best option. In contrast, in patients with clinically highly susceptible unstable angina, the use of imaging or coronary CCTA would be the best option (22). The 2019 ESC Guide noted that in patients with extensive CAD, CT-based FFR is not inferior to ICA and FFR in deciding and identifying vascular reconstruction targets (1). A 26-monthes follow-up study showed that CCTA is more discriminating than functional tests in predicting major adverse cardiovascular events (MACE) (23). For CCTA or invasive angiography stenosis, further evaluation using noninvasive or invasive functional tests may be necessary before extremely severe stenosis (diameter stenosis >90%) detected (15).

## 3 EAT and INOCA

### 3.1 EAT and CMD

EAT has a microcirculation that is share with the heart. Its unique properties include increased fatty acid metabolism and transcripts, as well as genes associated with inflammation and endothelial function. In physiological conditions, EAT have metabolic, thermal production (similar to brown fat), and mechanical (heart protection) properties, where mitochondrial brown fat uncoupling protein-1 (UCP-1)-positive small single atrial fat cells have been described in epicardial fat (24). The metabolic of EAT has been confirmed, while brown adipose tissue (BAT)-specific genes, such as UCP-1 and PRD1-BF1-RIZ1 homologous domain containing 16 (PRDM-16), were also found at high levels of expression in human EAT (8). The histological characteristics of EAT also resemble those of brown and beige adipocytes. Studies have shown that EAT is rich in saturated fatty acids with high protein content and has the strongest release and absorption capacity of free fatty acids (FFAs) compared to other visceral fat libraries (24, 25); this may be due to EAT containing mesenchymal cell-derived myocardial stem cells (9, 26). Physiological conditions enable FFAs to spread bidirectionally from epicardial fat to the myocardium along the concentration gradient, and the vascular active cytokines secreted by EAT allow FFAs to enter the coronary artery and influence coronary tension. Epicardial fat acts as a buffer to prevent excessive exposure to FFAs in the heart muscle. The oxidation of FFAs in the heart provides most of their energy. Therefore, metabolic disorders and FFA enrichment interact with the heart muscle.

CAD patients tend to have more brown fat than white fat. This is demonstrated by a significant decrease in the expression of brown fat-like genes (peroxidase proliferator activated receptor γ coactivator Factor 1α (PGC-1α), UCP-1 and PRDM-16 and by increased expression of white fat-like genes (insulin-like growth factor binding protein (IGFBP) 3 and homologous frame (HOX) C8 and 9) (8). In addition to increased reactive oxygen content and proinflammatory gene expression in EAT, this process also reduced plasma fatty acid clearance. Thus, EAT whitening and subsequent localized dysfunction may trigger vascular inflammation and coronary AS. But the mechanism of EAT white differentiation and produces inflammation remains unclear. Additionally, hypoxia and abnormal changes interact with EAT cytokines (such as TNF, IL-6 and FFAs) to alter atrial tissue conductance, accelerate fat decomposition, and play a role in AS at different stages. Vascular tension and remodeling, smooth muscle cell proliferation and migration, oxidative stress, congenital inflammatory response, and plaque instability are among them (8). In the early stages of these diseases, EAT acts as promoters of AS. This suggests that EAT might have a greater effect earlier than in the later stages of the disease. Many experts believe that thickened epicardial fat will cause physical obstruction of diastolic filling, which will lead to atrial enlargement, impaired ventricular diastolic filling, and ischemia (24). This may be a link between structure and dysfunction in the later stages of the disease.

EAT contributes to AS, and focal obstructive lesions occur in the coronary segments where there are the most dysfunctional electrophysiological properties adjacent to areas of thick epicardial fat. This inflammation can affect the structure and function of adjacent tissues through paracrine (27). In the Tomasz Mazurek et al. study, increased local inflammation in patients with severe CAD did not correlate with plasma circulating cytokine concentrations. It confirmed that bioactive molecules in peri coronary tissue may alter arterial homeostasis and the inflammatory cells in adipose tissue is only indicative of an approaching plaque rupture (28). This can only account for the interaction of CAD with inflammatory changes in EAT, but not for the sequence between them.

### 3.2 Mechanism of EAT causes INOCA

**Figure 2:**
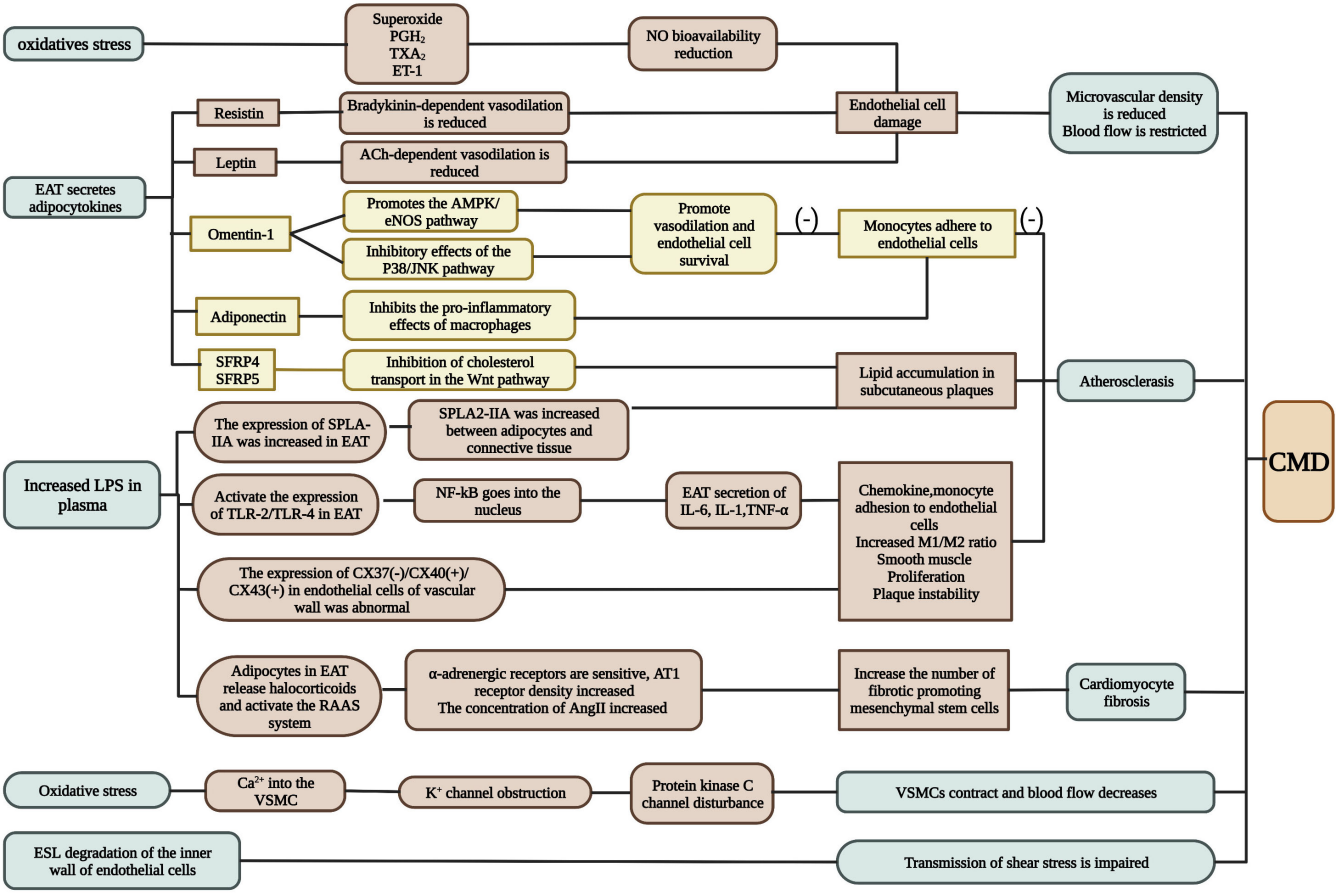
The mechanism of INOCA. INOCA is represented by CMD. It can be caused by oxidative stress, adipokines produced by EAT, changes in circulating factor concentrations in plasma, and changes in ESL. CMD is determined by a combination of factors including endothelial dysfunction, vascular smooth muscle cell hyperreactivity, vascular remodeling, vascular fibrosis, and changes in macrophage function (see text for details). A possible adjustment procedure is proposed for this. CMD: coronary microvascular dysfunction.*Green represents several conditions that produce CMD under abnormal EAT; brown represents pathways that promote inflammation; yellow represents pathways that inhibit inflammation. (Created with BioRender.com).

**Figure 3:**
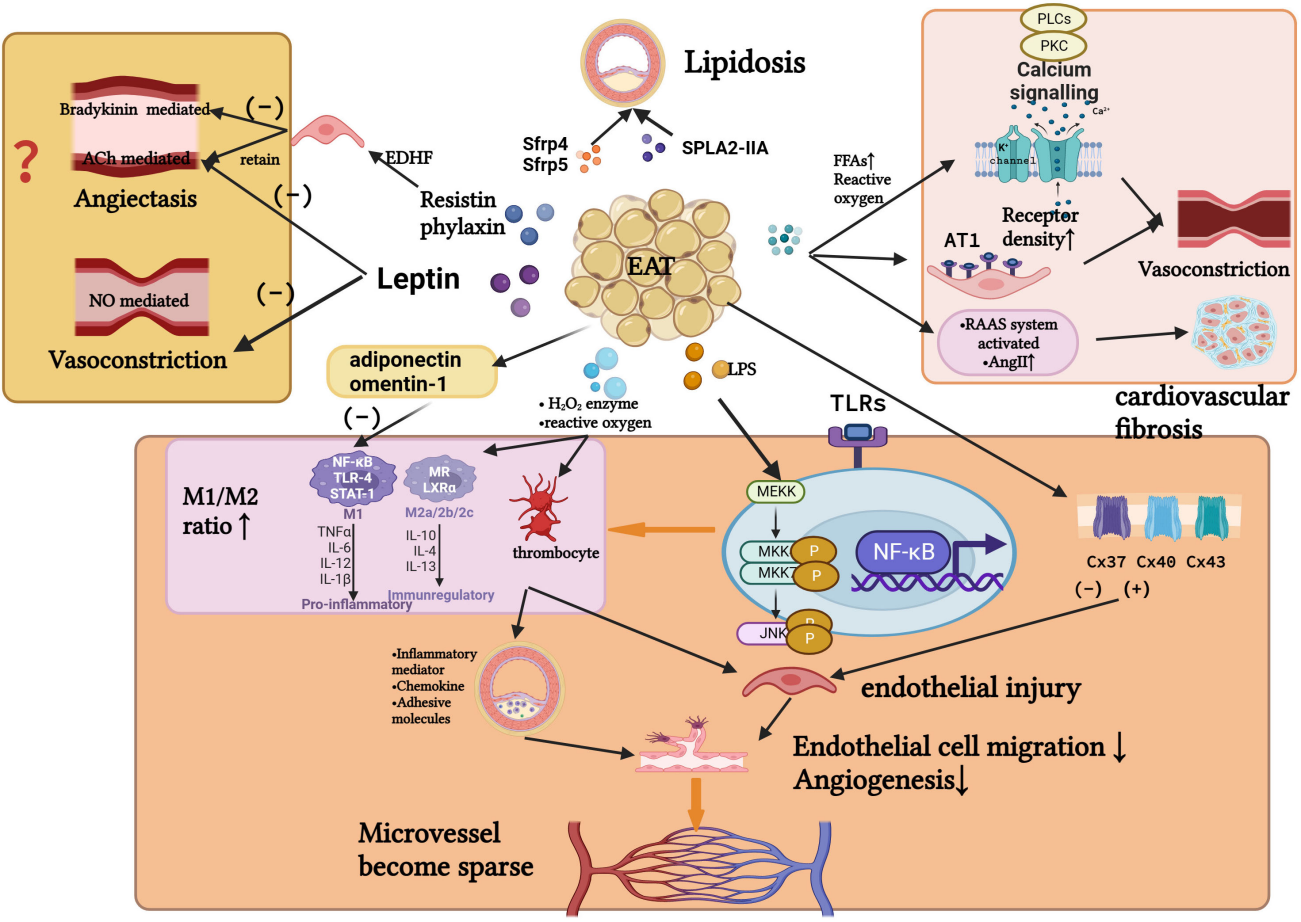
Several possible relationships between inflammatory EAT and the pathogenesis of INOCA. Inflammation causes EAT to secrete cytokines and adipokines, altering the function of the vascular endothelium and the phenotype of macrophages. In turn, it affects the function of vascular smooth muscle, the remodeling of microvascular density, deposition of subcutaneous plaque lipids, and the mesenchymal fibrosis. These may affect perfusion of myocardial blood flow, leading to the development of INOCA. (Created with BioRender.com).

At present, the mechanisms of INOCA are mostly believed to be endothelial injury and oxidative stress (**Figures 2**, **3**). Several studies have confirmed that INOCA occurs due to decreased microvascular density, mesenchymal fibrosis, contraction of smooth muscle cells, and deposition of subcutaneous plaque lipids. The local vascular environment is changed by EAT during inflammation, as it activates adipokines and proinflammatory factors. The relationship between EAT and CAD has been well established, but no studies have expressed the relationship between EAT and INOCA. Several mechanisms by which the inflammatory state of EAT may lead to INOCA are discussed below.

#### 3.2.1 Inflammation

CAD is a disease characterized by low-grade, chronic inflammation of the arterial wall. The early characteristics of the coronary microvascular response to inflammation are oxidative stress, reduced nitric oxide bioavailability, and endothelial activation. C-reactive protein is increased among subjects with microvascular angina compared with control subjects, further supporting a possible role of inflammation and endothelial dysfunction in causing CMD. CFR is reduced among patients with normal or minimally diseased coronary arteries and either systemic lupus erythematosus or rheumatoid arthritis, and prolonged systemic inflammation may also contribute to premature CAD in these patients (29).

#### 3.2.2 Endothelial function damage

When the endothelium is under stress, it may produce substances known as endothelin, thromboxane A_2_, prostaglandin H_2_ (30–33), and superoxide that restrict arterial blood flow. Net expansionary responses to various stimuli shift into contractile responses. Endothelin 1 (ET-1) is a potent vasoconstrictor of human coronary arteries. In microvascular angina (MVA)/vascular angina (VSA), Thomas J. et al. (31) used the ACh/adenosine thermodilution technique to confirm widespread endothelial damage. MVA and VSA were generally susceptible to ET-1. So, they (30) conducted another experiment in which the correlation of ET-1-related genes was investigated for 391 individuals with stable angina. A rs9349379-G allele of the ET-1 gene was independently associated with cardiomyopathy. In the population with G alleles, the likelihood of having high levels of ET-1 and myocardial dysfunction was more than twice (impaired myocardial perfusion, and impaired exercise endurance). As a result, inflammation may cause EAT to release ET-1 in CMD, thereby reducing NO bioavailability. Endothelial cell migrated, angiogenesis impaired, and angiogenesis and remodeling are unbalance, meaning that microvessel density decreases. It is unclear whether these changes are the results or the cause of CMD, so we assume they are reciprocal.

Indirectly or directly, EAT can also affect endothelial cells. Low-density lipoproteins (LDLs) are retained in the subcutaneous space by the enzyme secretory type II phospholipase A2 (SPLA2-IIA). In human atherosclerotic lesions, SPLA2-IIA expression was higher in epicardial fat (34). Expression of the human SPLA2-IIA gene in mouse models increased sensitivity to AS (35). EAT may cause lipogenesis by secreting SPLA2-IIA by its lipids into the stroma, resulting in conjugated fatty acids.

A transition from a resting to an active state is also involved in endothelial dysfunction. Several studies have shown that phenotypic transformation of macrophages can also cause endothelial dysfunction (25). Yoichiro Hirata’s team (36) confirmed that the level of proinflammatory cytokines in EAT of CAD patients negatively correlated with the level of anti-inflammatory cytokines. As the M1/M2 macrophage count increased with the Gensini score, it was evident that the M1/M2 macrophage count increased with CAD severity. A. R. Baker et al. (37) found that whole body lipopolysaccharide levels increased among patients with CAD. Additionally, TLR-2 and TLR-4 expression levels (markers of macrophage activation) were higher in EATs of CAD patients. Lipopolysaccharides trigger inflammation by activating TLRs (24) (**Figure 3**).

Additionally, connexins (a family of proteins), semi-channels and gap junction channels, have been shown to play a role in atherosclerosis (38). Nonetheless, in Cx40del mice, endothelial cells on the surface of advanced atherosclerotic lesions did not express CX40. Near lesions, endothelial cells not affected by lesions exhibited large amounts of Cx40 protein. It is suggested that Cx40 may be a marker of early endothelial injury.

#### 3.2.3 Adipokines regulation

EAT can release adipokines to regulate the progression of AS. Various studies have found (33) that resistin does not affect coronary blood flow, although it damages endothelial function. It is believed that resistin delays bradykinin-mediated vasodilation but permits ACh-mediated vasodilation. ACh-mediated vasodilation is decreased by leptin. Additionally, leptin inhibits contractile responses induced by AngII on aortic vascular smooth muscle cells (VSMCs) (9, 24). Intriguingly, EAT also expresses AngII and leptin. It is unclear whether EAT can affect the coronary endothelium *via* these factors, but it deserves further investigation.

The M1/M2 ratio of CAD patients was negatively correlated with the expression of adiponectin in EAT, but this correlation was not statistically significant (36). G. Iacobellis et al. (25) found that adiponectin levels near left coronary artery (LCA) were associated with the expression in EAT of CAD patients, whereas have no relationship with serum adiponectin levels. Yu Du et al. showed that adiponectin in the CAD group was lower than in the non-CAD group (39). Both stenosis and non-stenosis the difference was statistically significant in the CAD group. They also detected another adipokine of omentin-1. EAT omentin-1 level was markedly decreased in CAD patients compared with NCAD patients. In CAD patients with and without stenosis, omentin-1 ratios were 0.01:0.14 (P=0.0127) (39).

Wnt pathway-related proteins are highly expressed in AS lesions. It plays a major role in AS by transporting cholesterol and regulating inflammation. In the single-factor analysis of Q. Ji, Secreted frizzled-related protein 4 (Sfrp4) in EAT of CAD and non-CAD cardiac surgery subjects was a significant difference. Sfrp4 mRNA levels in EAT correlated with CAD, a multivariable linear regression model found that the association was independent of fasting glucose, age, and BMI (40). Sfrp5, which competitively binds with wingless-type family member 5a (Wnt5a), controls chronic inflammation, inhibits atherosclerosis, and represses metabolic conditions (41, 42). In our previous study, Wnt5a mRNA expression in EAT was found to correlate with the presence of CAD. Also, Sfrp5 mRNA and protein levels in EAT of CAD patients were shown to be reduced for the first time. And these inverse correlations were further confirmed after adjustment for other conventional risk factors (41). It means low Sfrp5 and high Wnt5a levels were associated with Chinese CAD patients.

#### 3.2.4 Neurohumoral regulation

Endothelial cells and the autonomic nervous system interact closely. Activation of β-adrenergic receptors in vascular smooth muscle cells causes vasodilation, activation of α-adrenergic receptors causes vasoconstriction, and activation of toxicological receptors causes vasoconstriction. Studies have confirmed that chronic metabolic disorders activate the RAAS system (33). The flow of AngII, combined with the AT1 receptors, significantly increase vasoconstriction. The sensitivity of alpha-adrenergic receptors also affects the coronary blood flow.

Ana M et al. found a correlation between EAT inflammatory, increased aldosterone and impaired vasodilation in obese people (43). An adrenal gland and adipose tissues over-synthesis corticosteroid signals, revealing a proinflammatory state in adipose tissue.

A study has shown that hypoxia causes microcirculation vessels to weaken to vasodilators, and this is due to cyclooxygenase (44). It was also observed that in the high-fat model, the impairment of upstream K_ATP_ channels was associated with impaired vasodilator response to acidosis (33). Inhibition of coronary arteriolar vasoconstriction by protein kinase C (via the K_ATP_ channel). Oxidative stress decreased the coronary response to ischemia-induced vasodilators. Therefore, changes in cardiac perfusion will likely occur after the development of oxidative stress and endothelial dysfunction.

#### 3.2.5 Emerging theory

Among endothelial cells, there is a membrane-bound layer of macromolecules (polysaccharide calyx) and another relatively thick layer of plasma components at the lumen surface, called the endothelial surface layer (ESL) (45). Accordingly, it hypothesized that CMD might be associated with lower arterial-mediated vasodilation of NO (4).The ESL regulates blood flow by transmitting shear stress (the transmission of shear force). ESL is very unstable and easily degraded by oxygen free radicals, ischemia, inflammation, or altered plasma composition.

## 4 EAT and the traditional risk factors affecting INOCA

### 4.1 EAT and metabolic disorders

EAT thickness is associated with metabolic syndrome in a significant and independent way, according to several clinical studies, it is independently on other cardiometabolic risk factors, such as insulin resistance, fasting glucose, CRP, liver enzymes, and carotid intima-media thickness (3, 24). On the other hand, some studies showing EAT were not correlated with traditional factors or AS (**Table 2**). Patients with CAD (48) had low mRNA levels of glucose transporter-4 (GLUT4) and abnormal levels of retinol binding protein 4 (RBP4). Cells in EAT release higher levels of RBP4 than subcutaneous fat cells. Thus, it is speculated that EAT may lead to locally adverse lipid and glucose spectra and insulin resistance. Studies have shown that (25) EAT is associated with an increase in serum transaminase levels and the incidence of hepatic steatosis in obese individuals. However, these changes are not related to obesity but to excessive visceral fat levels in these individuals. Due to increased FFA production and intrinsic insulin resistance, we conclude that epicardial and intrahepatic fat are directly linked. Nevertheless, Antonopoulos AS demonstrated that perivascular adipocyte lipid accumulation was positively correlated with atherosclerotic plaque load but not with systemic insulin resistance (49). It appears that perivascular adipocyte lipid accumulation is caused by local inflammation rather than systemic metabolic disorders (such as insulin resistance). A study by M. Shimabukuro et al. discovered that EAT volume is associated with coronary artery calcification independent of CAD (50). Interestingly, inflammatory cells in adipose tissue reflect only plaque rupture, in a similar manner to inflammatory infiltration of the outer membrane and surrounding blood vessels in advanced AS. Additionally, dipeptidyl peptidase-4 inhibitors have been shown to decrease EAT accumulation (27). However, it did not affect the anti-inflammatory properties or secretion of proinflammatory factors in EAT. In this case, insulin resistance is not the initiator, but the promoter of inflammation caused by EAT. Furthermore, adipose changes occurred before insulin resistance and CAD.

**Table 2:** Independent association of INOCA with traditional cardiovascular risk factors.

Author and Year	Sample size and feature	Methods	Traditional cardiovascular risk factors (Independent correlation)						
			Diabetes	BMI	Women	Hypertension	High cholesterol	Smoking	history of CAD
(5) Jaskanwal D. Sara, MBCHB 2015	Total 1498, CFRAdn+CBFAch+ 520 (47.8±11.9), CFRAdn+CBFAch-478 (49.8±12.6), CFRAdn-CBFAch+ 173 (52.8±13.7), CFRAdn-CBFAch-268 (53.9± 11.5)	Using an intracoronary Doppler guidewire, evaluating changes in CBF in response to acetylcholine	NO	YES	YES	NO	NO	YES	
(10) Shahar Lavi MD 2007	Total 881, Smokers (n=115) (43±1), Previous Smokers (n=314) (52±0.6), Never Smokers (n=452) (50±0.6)	ACh Testing	NO			NO	NO	YES	
(11) Peter Ong,MD 2014	921, Mean age (62±12)	ACh Testing	NO		YES	NO	NO	YES	YES
(29) Venkatesh L Murthy 2014	405 men Age 61.2 (52.8-68.8), 813 women Age 62.3 (54.1-71.6)	on rest/stress PET myocardial perfusion imaging, Coronary flow reserve	YES	YES	NO	YES			
(30) Thomas J Ford 2020	391, 151 INOCA [Genetic analysis 140, Mean age (61.1±10.1)]	interventional diagnostic procedure, undergo quantitative perfusion cardiac magnetic resonance (CMR) imaging at 1.5 T using pharmacological stress testing with intravenous adenosine (140mg/kg/min), Serum ET-1 was determined			YES	NO			
(16) Carl J. Pep, MD 2010	189 women, Mean age (55±10)	adenosine injected doppler flow testing, Inflammatory markers measured	NO	YES		YES	YES		
(46) Iida Stenström 2017	189, Mean age 62 (47–80)	coronary computed tomography angiography, quantitative 15O-water PET perfusion imaging, invasive coronary angiography	YES		YES	YES		YES	
(19) Louise E J Thomson 2015	118 women, Mean age (53.9±11.4)	Cardiac MRI, stress perfusion, invasive coronary reactivity testing (CRT)	NO			YES		YES	YES
(47) Jaime L Shaw 2018	22 females, Mean age 52.6 ± 13	native T1 mapping cardiac MR including function, stress/rest perfusion, and viability imaging.	YES			YES			

Interventional diagnostic procedure (IDP) that combined guidewire-based direct measurement of coronary vascular function followed by pharmacological vasoreactivity testing.

In patients with advanced CAD, there was no strong correlation between inflammatory signals from EAT and plasma inflammatory biomarkers (28). Similarly, circulating inflammatory biomarkers were not significantly associated with EAT concentrations. There was no significant relationship between inflammation caused by EAT in CAD patients and systemic inflammation (obesity, diabetes). It may therefore appropriate to speculate that the local inflammation caused by EAT is not associated with traditional risk factors.

### 4.2 Gender differences

Even though the prevalence of heart disease is higher in men than in women, the overall in-hospital mortality rate is higher than in men. This may be due to the atypical symptoms of heart attack in women (chest discomfort, weakness, dizziness) (51). Women in INOCA are approximately four times more likely than men to be readmitted to the hospital with ACS/chest pain within 180 days (3). It shows that gender difference is a unique characteristic of INOCA, women for its susceptible population. Several studies have found that women with CMD have higher myocardial triglyceride content, which may translate into ectopic fatty acid deposition in cardiomyocytes, resulting in myocardial mechanical damage in the diastolic period (3). Estrogen might control *in vivo* vasodilation by inducing cardiomyocyte hyperpolarization, altering ATP-sensitive potassium channel dynamics in myocytes, and inhibiting Ca^2+^ and ET-1 controlled contractions of cardiomyocytes. At the same time, estrogen can induce cardiomyocyte-mediated endothelin-independent vasodilation by stimulating prostacyclin production (52). Since adipocytes, immune cells, and mitochondria express estrogen receptors specifically, and free fatty acid metabolism in women is more complex than in men. Estrogen receptors are expressed on the surface of immune cells and modulate immune activity. β-Estradiol inhibits immune activity by inhibiting the differentiation of proinflammatory helper T cells and M1 macrophages. Estrogen is also a determinant of adipose tissue metabolism and function. In a mouse model (53), estrogen has been shown to prevent impaired glucose tolerance and obesity by regulating adipogenesis and decomposition. Estrogen can also increase the expression of this gene through estrogen receptor-α in the mitochondria of brown adipose tissue and affect mitochondrial production (3, 54). Studies have shown (8) that visceral adipose tissue (VAT) includes less insulin-sensitive tissue, and inflammatory cytokines and immune cells in the infiltrating blood vessels express more glucocorticoids and androgen receptors. VAT has been proven to be more sensitive than subcutaneous adipose tissue (32), and male fat is easily deposited in VAT due to its unique composition of androgens. FFAs is first deposited in female cardiomyocytes by the expression of estrogen receptors. Studies have shown that myocardial dysfunction is independently associated with metabolic syndrome, and men are at greater risk than women (54). In INOCA, however, females are more prevalent than males and EAT thickness in women was associated with more risk factors (32, 55, 56). Therefore, is it possible to speculate that obstructive CAD occurs after INOCA? Are women more inclined to have INOCA due to diffuse lipid deposition compared to men who are prone to focal obstruction? Further researches are needed.

## 5 EAT detection

**Figure 4:**
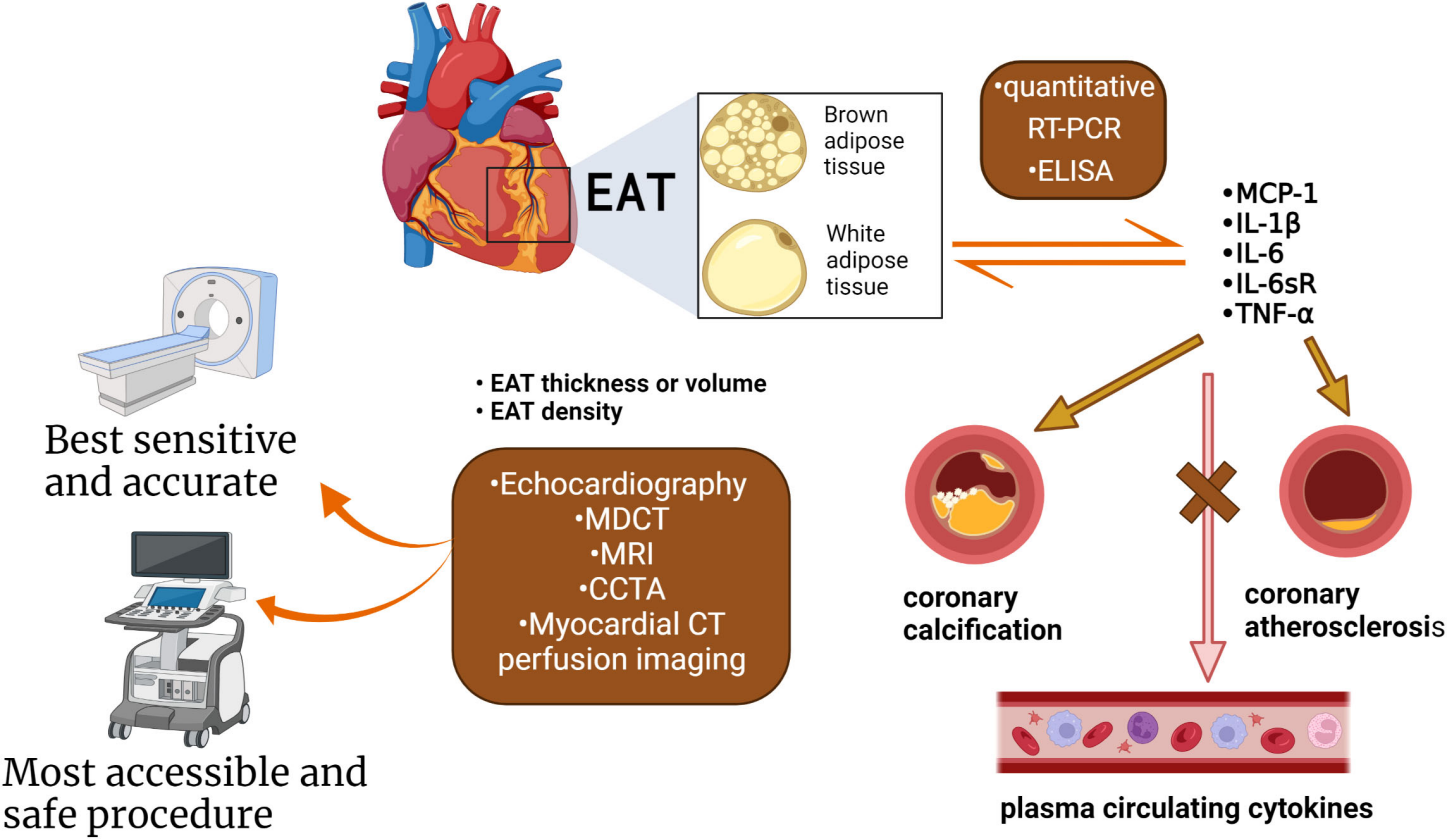
The methods used to measure the quantity and quality of EAT. Quantification of EAT has seen improvements over the years attributed to the advancements in non-invasive imaging techniques: CT, as well as CMR, PET. Evaluation indicators as thickness, volume, and density. EAT under chronic inflammation shifts from brown fat to white fat. The quality of the EAT was changed, such as inflammatory status, the type of adipokines, and microRNA. The cytokines and adipokines within the EAT can be measured by quantitative RT-PCR and ELISA. Cytokines correlate with the presence of CAD, severity, and coronary artery calcification, but not with circulating cytokines in plasma. EAT inflammation was independent of several clinical variables (obesity, diabetes, or chronic therapy with statins or ACE inhibitors). (Created with BioRender.com).

Researchers have confirmed that the quantity and quality of EAT are both associated with AS in CAD patients by different ways (**Figure 4**; **Table 3**). Its thickness, a noninvasive echocardiographic assessment, proved to be the best independent predictor of carotid IMT (8). 5.0 mm thick EAT in diastolic identified a higher likelihood of vascular disease in the presence of carotid plaque (57). EAT was positively associated with coronary artery calcium (CAC) in the study, even after adjusting for other CAD risk factors and BMI (58). EAT is also closely associated with the presence of “soft” or “mixed” vulnerable plaques and is associated with negative outcomes (59). Moreover, there was a weak correlation between EAT and the Framingham risk score (57) and EAT thickness (>3.0 mm) was an independent predictor of CAD. In the same cohort, a 4.5 mm definition of EAT thickness identified five elements of metabolic syndrome, with 9.5 mm and 7.5 mm predicting metabolic syndrome in men and women, respectively. EAT thickness of 8.0 mm and 7.1 mm (men and women) indicate abnormal fasting glucose (60). A higher value is more predictive of heart disease after systemic metabolic disorders. However, in a study of middle-aged subjects with suspected metabolic syndrome (54), the thickness of EAT was associated with hs-CRP level, left ventricular mass, and subclinical myocardial dysfunction only in men. This suggests that the inflammatory activity of EAT induces myocardial remodeling and dysfunction in middle-aged subjects but is attenuated in female subjects. Therefore, it is speculated that the thickness of EAT may be a marker of the early process of cardiac remodeling and dysfunction in these patients.

**Table 3:** Comparison of methods for detecting EAT.

Method	Index	Standard	Advantages	Disadvantages
MRI	thickness/ volumetric	the greatest amount of epicardial fat can be found at the lateral right ventricular wall, using the summation of slices method	gold standard, radiation-free, not limited by the position and orientation of the imaging planes	Costly, time-intensive
CT imaging*	thickness/ volumetric	performed along the right ventricular anterior free wall in a single sagittal slice, ranging -190 to -30 Hounsfield units	higher spatial resolution, reproducibility, best visibility of the pericardium	Costly, radiation exposure
Via transthoracic echocardiographic ultrasound	thickness	on the free wall of the right ventricle, normal values: around 5mm	relatively inexpensive, widely available	linear measurement, low reproducibility
EAT attenuation as measured by CT*	mean attenuation	EAT attenuation was calculated as mean Hounsfield units of all pixels ([-190, -30]HU)	markers of adipose tissue inflammation	
FDG-PET/computerized tomography*	EAT inflammatory activity	analysis differences between SUV in different locations	reflect glucose metabolism of the tissue	

CT computed tomography, FDG-PET 18-fluorodeoxy glucose -positron emission tomography, SUV Maximal standardized uptake value

*no meta-analysis has yet defined normal and pathological values

EAT density detected by echocardiography, magnetic resonance imaging (MRI) and PET/CT may be used as a biomarker for early coronary atherosclerosis, as it is an explanation for EAT composition and its effect on CAD (8, 61). The radioactive density of adipose tissue detected by CT is associated with differences in adipose tissue composition, which may promote the development of coronary atherosclerosis independent of the volume of EAT. The relationship between EAT attenuation and CAC distribution and density was not affected by gender (61). In men with severe CAD, lower CT attenuation at EAT was associated with higher CAC (14), independent of EAT volume and BMI. Interestingly, the attenuation of EAT was independent of the distribution range of calcium in the four major coronary arteries but related to the calcium density of each plaque (61). This may indicate that EAT may not be an instigator of CAC development but merely a catalyst for the rupture of stable atherosclerotic plaques.

## 6 Conclusions and outlook

INOCA has now entered the public eye for its undervalued cardiogenic deaths. Its microcirculation system is like the coronary artery, which is also a research hotspot. In recent studies, it has been found that the EAT calcification fraction is independently correlated with EAT thickness. However, some studies have confirmed that EAT is not correlated with traditional risk factors. As CAD deterioration, inflammation of the coronary system and microcirculation are isolated from systemic inflammatory changes. The high sensitivity of EAT relative to subcutaneous fat makes it possible to detect early dysfunction of the coronary artery and microcirculation system. In conclusion, maybe EAT could be a goalkeeper for INOCA, and more reliable clinical research and basic experiment are needed.

## Author contributions

TS contributed to the conception of the review. ZS wrote the first draft of the manuscript. WH wrote some sections of the manuscript. ZM and TS wrote and modified the review.Both authors have reviewed the final version of this manuscript before submitting it to topic editors of the journal.
